# Camera traps are an effective tool for monitoring insect–plant interactions

**DOI:** 10.1002/ece3.8962

**Published:** 2022-06-02

**Authors:** Qaim Naqvi, Patrick J. Wolff, Brenda Molano‐Flores, Jinelle H. Sperry

**Affiliations:** ^1^ Department of Natural Resources and Environmental Sciences University of Illinois Urbana Illinois USA; ^2^ Construction Engineering Research Laboratory, Engineer Research and Development Center U.S. Army Corps of Engineers Champaign Illinois USA; ^3^ Illinois Natural History Survey University of Illinois Champaign Illinois USA

**Keywords:** Camera trap, detection, digital video recording, game camera, insect monitoring, pollination, pollination biology, pollinator monitoring

## Abstract

Insect and pollinator populations are vitally important to the health of ecosystems, food production, and economic stability, but are declining worldwide. New, cheap, and simple monitoring methods are necessary to inform management actions and should be available to researchers around the world. Here, we evaluate the efficacy of a commercially available, close‐focus automated camera trap to monitor insect–plant interactions and insect behavior. We compared two video settings—scheduled and motion‐activated—to a traditional human observation method. Our results show that camera traps with scheduled video settings detected more insects overall than humans, but relative performance varied by insect order. Scheduled cameras significantly outperformed motion‐activated cameras, detecting more insects of all orders and size classes. We conclude that scheduled camera traps are an effective and relatively inexpensive tool for monitoring interactions between plants and insects of all size classes, and their ease of accessibility and set‐up allows for the potential of widespread use. The digital format of video also offers the benefits of recording, sharing, and verifying observations.

## Graphical abstract

Declines in insect and pollinator populations have led to a need for high‐quality, accessible monitoring. We found that off‐the‐shelf, close‐range game cameras can significantly improve insect‐plant monitoring.

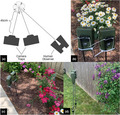

## 1 INTRODUCTION

The health and diversity of insect populations are major contributing factors to global food production, ecosystem function, and economic stability (Gallai et al., 2009; Ollerton et al., 2011; Steffan‐Dewenter et al., 2005). Due to national and public concern over population declines, there has been a growing interest in insect and pollinator monitoring to inform possible proactive management strategies (Biesmeijer et al., 2006; Breeze et al., 2021; Potts et al., 2010; Steffan‐Dewenter et al., 2005). Good management requires good data, and monitoring is only useful if the data gathered are meaningful and of high quality (Kosmala et al., 2016; Kremen et al., 2011). Traditional methods of monitoring insect–plant interactions typically entail researcher observation at one or several focal plants over a set time period (e.g., 5–10 min, 1–4 h; Fitch & Vaidya, 2021; Kunin, 1993; Roy et al., 2016). However, human observations are prone to misidentification, a lack of verifiable proof in the form of pictures or video, and difficulty identifying insects to a relevant taxonomic level (Kremen et al., 2011; Roy et al., 2016).

Automated camera systems have been found to be a powerful yet underutilized tool for gathering large amounts of high‐quality insect data (Gilpin et al., 2017; Lortie et al., 2012; Pegoraro et al., 2020; Steen, 2017). Camera traps have been used to address numerous research questions including pollinator diversity and behavior (e.g., Howard et al., 2021; Manetas & Petropoulou, 2000) and insect predation (Grieshop et al., 2012). However, many of these camera systems use continuous video recording, which produces massive quantities of data, and require adaptations to achieve the desired recording settings or to extend battery life (e.g., Droissart et al., 2021; Lortie et al., 2012; Micheneau et al., 2008; Pegoraro et al., 2020; Steen, 2017). While these more sophisticated, custom systems have been shown to be effective (e.g., Droissart et al., 2021), an evaluation of a more user‐friendly, accessible, “out‐of‐the‐box” solution, such as game cameras, would benefit the less technologically savvy practitioner (Droissart et al., 2021; Steen, 2017).

Here, we evaluated the use of high‐definition, commercially available game cameras with close‐focus functionality to monitor insect–plant interactions and behaviors. We then compared the results to traditional human observations. Based on previous studies, we predicted cameras would capture more insect detections than humans in general (Lortie et al., 2012; Pegoraro et al., 2020). However, we further compared two recording settings available on most models of game cameras: scheduled and motion‐activated. We predicted the number of detections by cameras would vary based on insect body size. For example, motion‐activated cameras would capture more large insects because they would trigger the motion sensor, whereas the scheduled cameras make no use of the motion sensor feature. In addition, we evaluated insect detections by the two camera settings and human observations based on insect behaviors. We did not limit documented behaviors only to pollination behaviors in order to maximize the utility of the technology to answer a variety of research questions such as monitoring of insect diversity and/or interspecific interactions (Morse, 1986; Reed, 1995; Robertson & Maguire, 2005). We predicted that behaviors that result in more time at the flower (e.g., flower probing) would be detected similarly between both cameras and humans, but that fast‐moving behaviors (e.g., flying) would be more likely to be detected by cameras. Our goal is to present a proof‐of‐concept for high‐precision insect–plant monitoring that uses relatively inexpensive tools, requires minimal training to carry out, and can be used to address a wide variety of insect‐plant research questions.

## 2 METHODS

### 2.1 Insect monitoring

We conducted a series of 16 paired human and camera trap observation trials to compare game cameras (hereafter, “camera traps”) to human observers for documenting insect–plant interactions. Trials took place at five study sites in Champaign County and DuPage County, Illinois, U.S.A., between 1 July and 21 August 2020. Each site was a suburban or exurban residential property containing numerous planted native and ornamental species.

**FIGURE 1:**
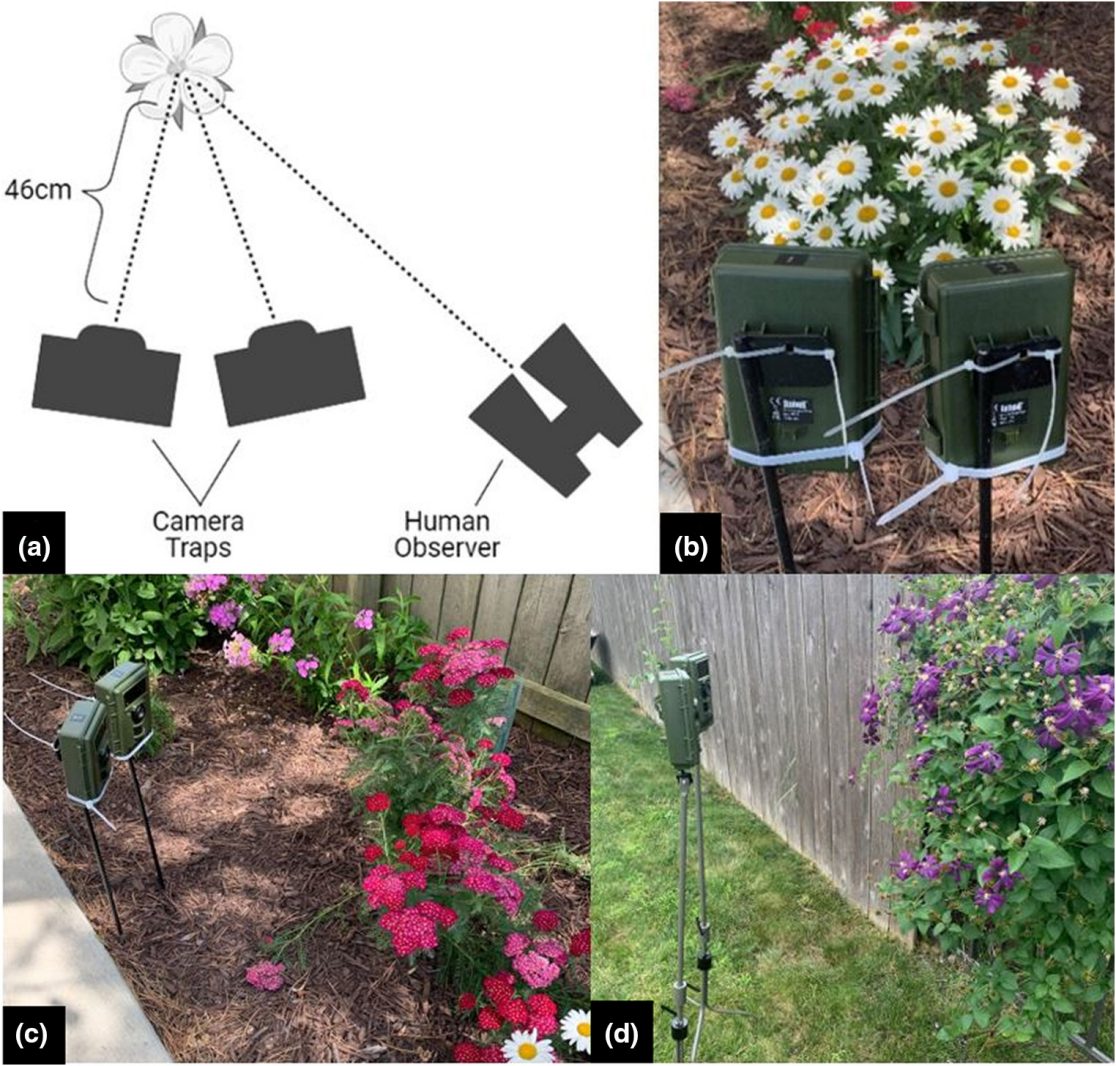
Image (a) shows a schematic of insect observation trials. Two camera traps with different recording settings (one scheduled, one motion‐activated) were placed side‐by‐side on separate tripods, 46 cm from the focal flower. A human observer viewed insect–plant interactions occurring on the focal flower using binoculars. Images (b–d) display the camera trap setup with a variety of lighting conditions flower types. Each trial lasted 3 h and took place in Champaign County and DuPage County, Illinois, USA

For each trial, we deployed two cameras (Bushnell NatureView HD model 119740, Bushnell Outdoor Products, Overland Park, Kansas), each equipped with a close‐range 460 mm lens. Cameras with similar specifications are available from alternate manufacturers (e.g., Reconyx Professional Series cameras with custom focal distance, Reconyx, Holmen, Wisconsin). One camera was set to record one 60‐s‐long video every 5 min (hereafter, “scheduled” camera) and the other was set to record one 60‐s‐long video when triggered by motion (hereafter, “motion‐activated” camera) (See Supporting Information S2 for video examples). Cameras were mounted on separate tripods and aimed at a focal flower or small cluster of flowers of the same species at a distance of 46 cm (Figure 1). We used the handheld Live View accessory provided with the camera to ensure proper focus and framing of the focal flower part. After activating the pair of cameras, an observer sat 1–2 m from focal flower and observed insect interactions using binoculars, in order to facilitate more accurate insect identification from a distance. Although the majority of pollinator observations are conducted with the naked eye, we found binoculars to be a helpful tool when focusing on one or a few focal flowers in order to compare it to the performance of camera traps. The observer monitored the focal flower for 3 h after activating the cameras and recorded the identity, quantity, and behavior of all insects interacting with the flower. All analyses were conducted with data collected during a 3‐h period in which observers and cameras simultaneously monitored the focal points of the plant.

Insect monitoring was conducted using 13 plant species, covering a range of flower morphologies, and included native and nonnative species (Table 1). Flower species diversity was favored over replication in the experimental design in order to display game cameras’ performance when compared to humans in a variety application cases. Observations were conducted during daylight hours between 08:57 and 18:14. Trials were not conducted in inclement weather with precipitation or during storms.

**TABLE 1:** Number of insect–plant interactions detected on each species of plant monitored during 16 3‐h observation trials in Champaign County and DuPage County, Illinois, USA

Focal plant species	Origin	IL County	Trials	Scheduled	Motion	Human	Total
*Asclepias tuberosa*	Native	Champaign	1	77	3	22	102
*Coreopsis* sp.	Native	DuPage	1	7	0	4	11
*Daucus carota*	Non‐native	DuPage	1	29	0	45	74
*Echinacea paradoxa*	Non‐native	Champaign	3	432	408	118	958
*Echinacea purpurea*	Native	Champaign	1	9	2	24	35
*Eutrochium purpureum*	Native	DuPage	1	199	0	65	264
*Hyssopus officinalis*	Non‐native	Champaign	2	1920	1235	217	3372
*Mentha spicata*	Non‐native	DuPage	1	150	284	322	756
*Monarda fistulosa*	Native	DuPage	1	23	7	19	49
*Nepeta mussinii*	Non‐native	DuPage	1	104	49	62	215
*Rudbeckia hirta*	Native	Champaign	1	46	9	54	109
*Verbena urticifolia*	Native	Champaign	1	132	8	64	204
*Veronica* sp.	Native	Champaign	1	350	15	23	388
Total			16	3478	2020	1039	6537

### 2.2 Video annotation

After the observation trials, camera trap videos were reviewed and annotated using the image analysis software Timelapse 2 (Greenberg, 2021). For each video, a trained observer recorded the identity, quantity, and behavior of all insects present. In most cases, the observer who conducted the plant observations also annotated the videos for that trial. Insects were identified taxonomically order level Diptera, Formicidae, Coleoptera, Lepidoptera, Hemiptera, Hymenoptera excluding the family Formicidae, or unknown. Formicidae were considered separately from the rest of Hymenoptera due to its morphological and behavioral differences. Hymenoptera was further organized into three size categories (small, medium, large) according to body length. The small size category represents insects approximately less than 10 mm in length (e.g., *Chrysis* spp., *Agapostemon* spp.), medium size insects are approximately 10–15 mm in length (e.g., *Vespula* spp., *Anthidium* spp.) and the large insects are approximately greater than 15 mm in length (e.g., *Bombus* spp., *Xylocopa* spp.). Because of variation in life history stages, we categorized Lepidoptera as either adult or larva rather than by size class. Insect behavior was classified into six behaviors: flying, hovering, landing, walking, probing, and moving between flowers. Insect interactions with leaves, stems, and other parts of the focal plant were ignored, as were interactions with flowers that were out of focus or in the background. Insects that were unidentifiable to taxonomic order were included in overall insect counts but excluded from order‐specific analyses.

### 2.3 Statistical analyses

All statistical analyses were conducted in Program R v. 4.0.0 (R Core Team, 2020). We created generalized linear mixed models to evaluate the influence of observation method (scheduled camera, motion‐activated camera, and human observer) on the number of insects of each taxonomic order detected during observation trials. We included trial number as a random effect to account for variability due to different observers and focal plant species among trials. Because the camera data were not continuous throughout the 3‐h observation period, we cannot directly compare results for individual detections but, rather, we compare overall differences in numbers and diversity of insects detected among methods.

We constructed our model set using the “lme4” package (Bates et al., 2015). We checked for overdispersion using the function by Bolker et al. (2021) and for zero‐inflation using the “performance” package (Lüdecke et al., 2021). Overdispersion was detected in many of our count data models (variance‐to‐mean ratios >2), thus we used a negative binomial distribution (White & Bennetts, 1996) for all models to maintain consistency and comparability among the model set. In cases where an individual model was not overdispersed, use of either a negative binomial distribution or Poisson distribution did not quantitatively and qualitatively affect model results (see Supporting Information S1 for all model summaries and diagnostic tests). We report odds ratios to quantify the effectiveness of each camera type relative to the traditional human observer method. To present odds ratios in a forest plot, we used the packages “ggplot2” (Wickham, 2016) and “ggforestplot” (Scheinin et al., 2021).

We compared the diversity of insects (number of taxonomic orders) detected by the three observation methods using a linear mixed model via the “lme4” package (Bates et al., 2015), again with trial as a random effect. We examined residual plots to confirm that assumptions of linearity and homoskedasticity were not violated. Next, we evaluated the influence of camera type (scheduled versus motion‐activated) on the body size of insects detected by creating generalized linear mixed models using a Poisson distribution. We compared detections of small‐, medium‐, and large‐bodied Hymenoptera, as well as larvae and adult Lepidoptera, between the two camera types. Size classes were not documented by human observers during trials, so we could not evaluate body sizes detected by humans in comparison to cameras. Last, we tested for differences in the frequencies that behaviors were observed by scheduled cameras, motion‐activated cameras, and human observers using chi‐square tests.

## 3 RESULTS

During 16 insect observation trials, scheduled cameras triggered on average 34.1 times per trial (median = 34.5 triggers, range = 27–36 triggers), resulting in 3478 insect detections (median = 121 detections, range = 8–1044 detections) in 545 min of video recordings (Table 1). Motion‐activated cameras triggered on average 16.3 times per trial (median = 7.5 triggers, range = 0–83 triggers), resulting in 2020 insect detections (median = 13.5 detections, range = 0–736 detections) in 261 min of video recordings. Human observers recorded 1039 insects (median = 45.5 detections, range =4–328 detections) in 2880 min of observation. The number of triggers on the scheduled camera was lower than the expected 36 triggers per trial due to equipment malfunctions. A total of 384 insects (11% of detections) were not identifiable to order from scheduled cameras, 127 (6% of detections) from motion‐activated cameras and 16 (2% of detections) from human observers.

**FIGURE 2:**
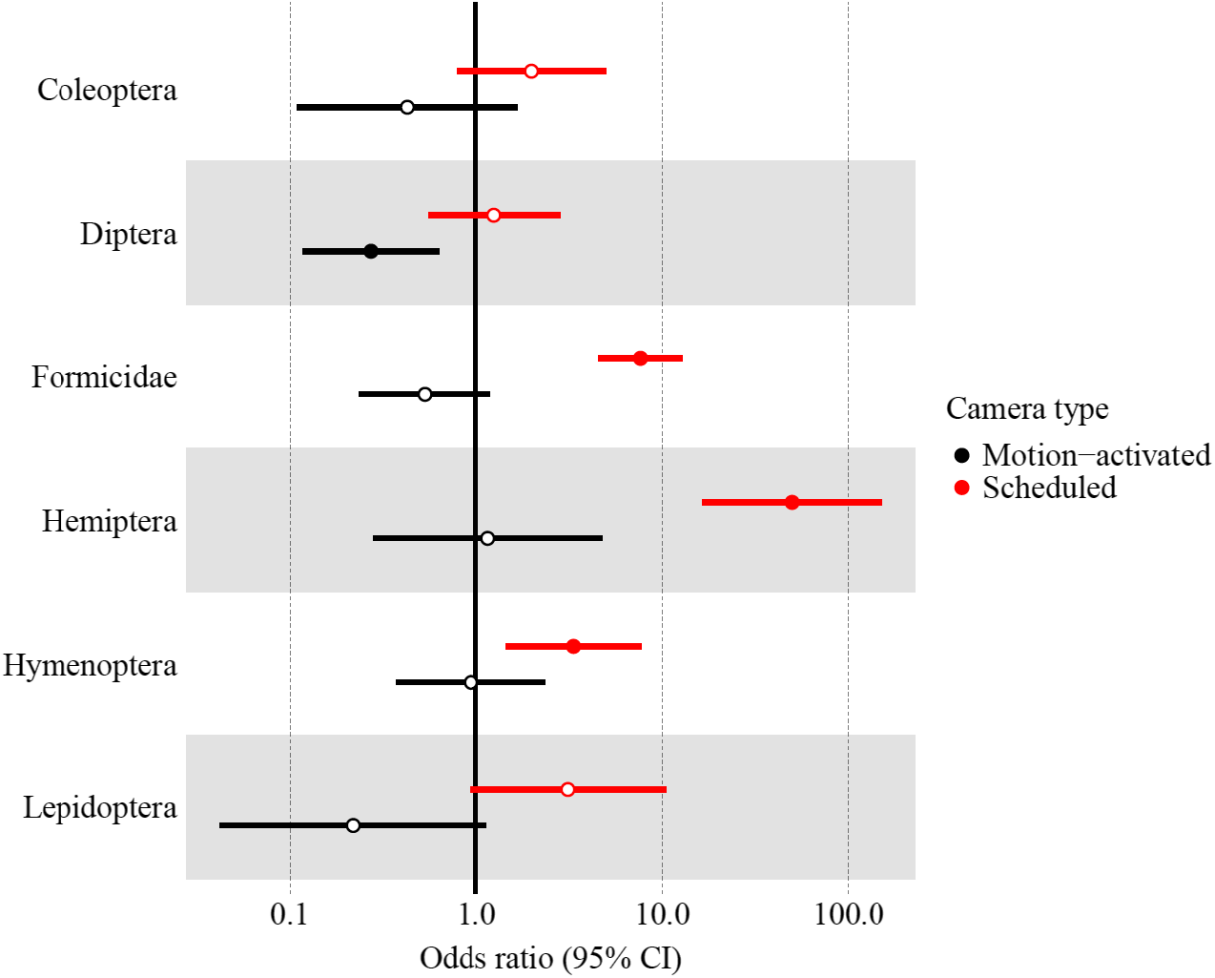
Odds ratios (dots) with 95% confidence intervals (lines) for the number of insect detections by motion‐activated cameras (black) and scheduled cameras (red) relative to human observers (vertical black line at odds ratio = 1), by insect order. Filled‐in dots indicate significant relationships compared to human observers. Hollow dots indicate relationships that are not significantly different from human observers. Odds ratios represent the number of times as likely the camera was to detect a given insect order compared to human observers during 16 3‐h observation trials in Champaign County and DuPage County, Illinois, USA. Note the *x*‐axis is on the log scale

Scheduled cameras detected more insects of all orders than motion‐activated cameras and human observers (Table 2). Compared to human observers, scheduled cameras were 3.35 (95% CI = 1.45–7.76) times more likely to detect Hymenoptera, 7.66 (95% CI = 4.71–13.28) times more likely to detect Formicidae, and 49.79 (95% CI = 16.35–151.65) times more likely to detect Hemiptera (Figure 2). Motion‐activated cameras were 0.27 (95% CI = 0.12–0.63) times as likely as human observers to detect Diptera (Figure 2). There were no significant differences between either camera type and humans at detecting Coleoptera or Lepidoptera (Figure 2).

**TABLE 2:** Number of insect‐plant interactions (mean ± SE) of six insect groups detected by scheduled cameras, motion‐activated cameras, and human observers during 16 3‐h observation trials in Champaign County and DuPage County, Illinois, USA

Order	Scheduled camera	Motion‐activated camera	Human observer
Coleoptera	0.88 ± 0.57	0.19 ± 0.19	0.44 ± 0.26
Diptera	20.56 ± 6.36	5.94 ± 2.67	14.88 ± 3.99
Formicidae	8.12 ± 4.14	0.56 ± 0.45	1.06 ± 0.60
Hemiptera	15.50 ± 15.10	0.31 ± 0.25	0.25 ± 0.19
Hymenoptera	207.63 ± 75.29	112.13 ± 52.30	48.25 ± 19.76
Lepidoptera	7.75 ± 5.52	0.38 ± 0.27	1.44 ± 0.67

The diversity of insect groups detected did not differ between human observers and scheduled cameras (β = 0.31, SE = 0.31, *p* = .326). However, motion‐activated cameras significantly underperformed both human observers (β = −0.94, SE = 0.31, *p* = .005) and scheduled cameras (β = −1.25, SE = 0.31, *p* < .001), detecting approximately one fewer insect order per trial. Scheduled cameras detected significantly more insects of all body size classes than motion‐activated cameras (all *p* < .01; Table 3).

**TABLE 3:** Number of insect–plant interactions (mean ± SE) of different size/life history stage classes of two orders of insects detected by scheduled and motion‐activated cameras during 16 3‐h observation trials in Champaign County and DuPage County, Illinois, USA

Pollinator type	Scheduled	Motion‐activated	*p*‐value
Large Hymenoptera	3.19 ± 1.33	1.25 ± 0.70	.0004
Medium Hymenoptera	40.50 ± 18.63	24.69 ± 11.52	<.0001
Small Hymenoptera	163.94 ± 62.32	86.19 ± 43.66	<.0001
Adult Lepidoptera	1.31 ± 0.70	0.13 ± 0.09	.0015
Larval Lepidoptera	6.44 ± 5.00	0.25 ± 0.19	<.0001

Note

*p*‐values indicate the significance of the fixed effect of camera type in generalized linear mixed models.

**FIGURE 3:**
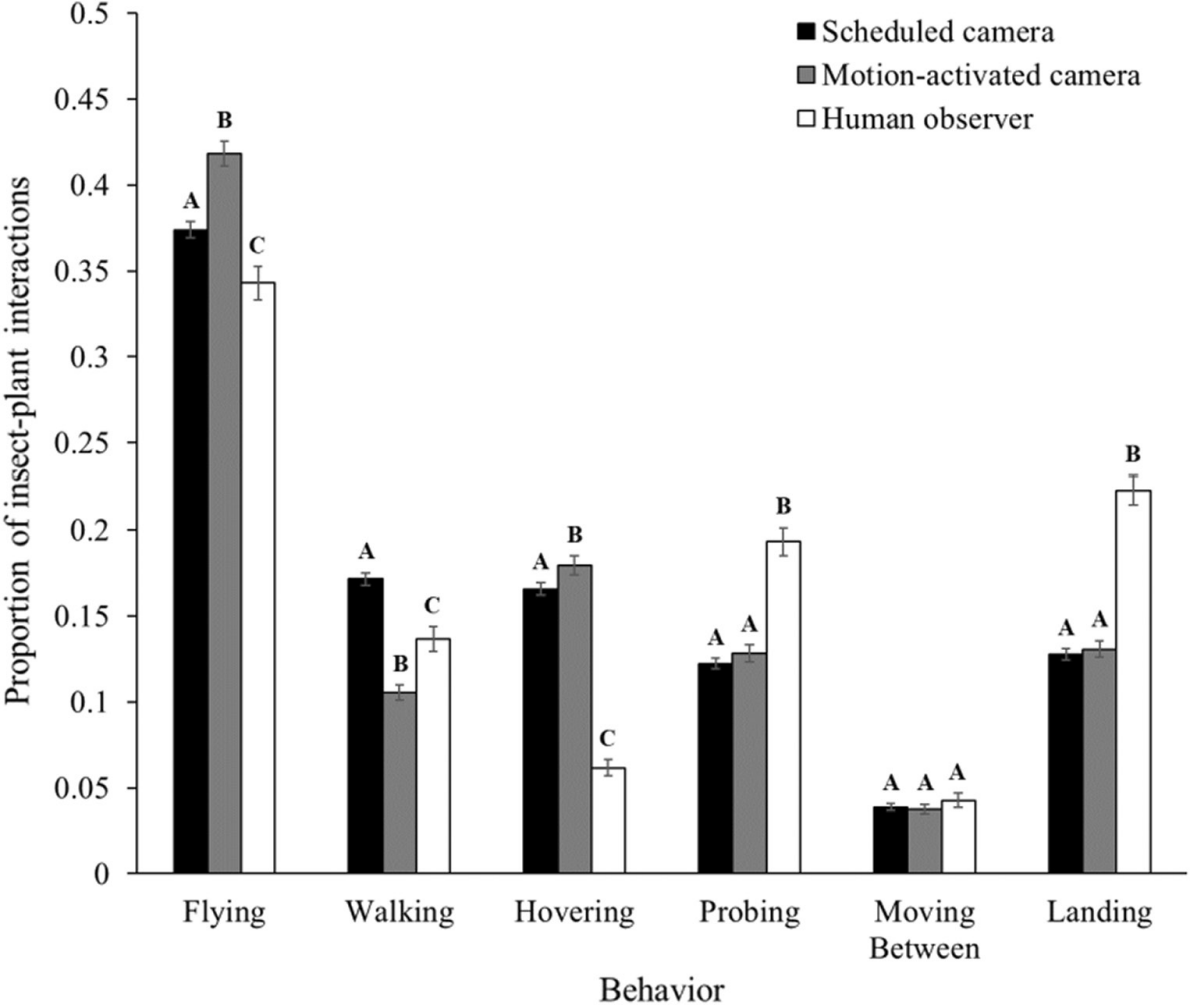
Proportion of insect–plant interactions in which insects performed six behaviors: flying, walking, hovering, probing, moving between flowers, and landing. Within a behavior, bars with different letters indicate significant differences among scheduled cameras (*n* = 10 520), motion‐activated cameras (*n* = 4462), and human observers (*n* = 2365)

Scheduled cameras documented 10 520 occurrences of six insect behaviors; motion‐activated cameras documented 4462 occurrences, and human observers documented 2365 occurrences (Figure 3). Overall, the three observation methods differed in their detection of behaviors (χ^2^ = 487.3, DF = 10, *p* < .0001). Compared to camera methods, humans recorded disproportionately more probing (human–scheduled camera: χ^2^ = 82.7, DF = 1, *p* < .0001; human–motion‐activated camera: χ^2^ = 62.0, DF = 1, *p* < .0001) and landing (human–scheduled camera: χ^2^ = 141.3, DF = 1, *p* < .0001; human–motion‐activated camera: χ^2^ = 97.9, DF = 1, *p* < .0001). Conversely, humans documented disproportionately less flying (human–scheduled camera: χ^2^ = 7.9, DF = 1, *p* = .0050; human–motion‐activated camera: χ^2^ = 37.4, DF = 1, *p* < .0001) and hovering (human–scheduled camera: χ^2^ = 28.6, DF = 1, *p* < .0001; human–motion‐activated camera: χ^2^ = 181.1, DF = 1, *p* < .0001). Compared to scheduled cameras, motion‐activated cameras recorded a higher proportion of flying (χ^2^ = 27.0, DF = 1, *p* < .0001) and hovering (χ^2^ = 4.4, DF = 1, *p* = .0359), but less walking (χ^2^ = 110.0, DF = 1, *p* < .0001). All three observation methods documented insects moving between flowers in similar proportions (scheduled camera–motion‐activated camera: χ^2^ = 0.1, DF = 1, *p* = .7285; human–scheduled camera: χ^2^ = 0.7, DF = 1, *p* = .3883; human–motion‐activated camera: χ^2^ = 1.0, DF = 1, *p* = .3082).

## 4 DISCUSSION

Our results demonstrate that commercially available game camera traps are an effective alternative to human observations for documenting insect–plant interactions and insect behaviors. In particular, cameras set to automatically capture video on a set schedule provided significantly higher numbers of detections of Formicidae, Hemiptera, and Hymenoptera than humans, and detected more insects of all sizes than motion‐activated cameras. Numerous studies have demonstrated the utility of modified cameras for documenting insect behavior and diversity (Grieshop et al., 2012; Howard et al., 2021; Manetas & Petropoulou, 2000). Our results suggest that even commercially available, unmodified cameras can collect valuable data for insect conservation and management goals.

**FIGURE 4:**
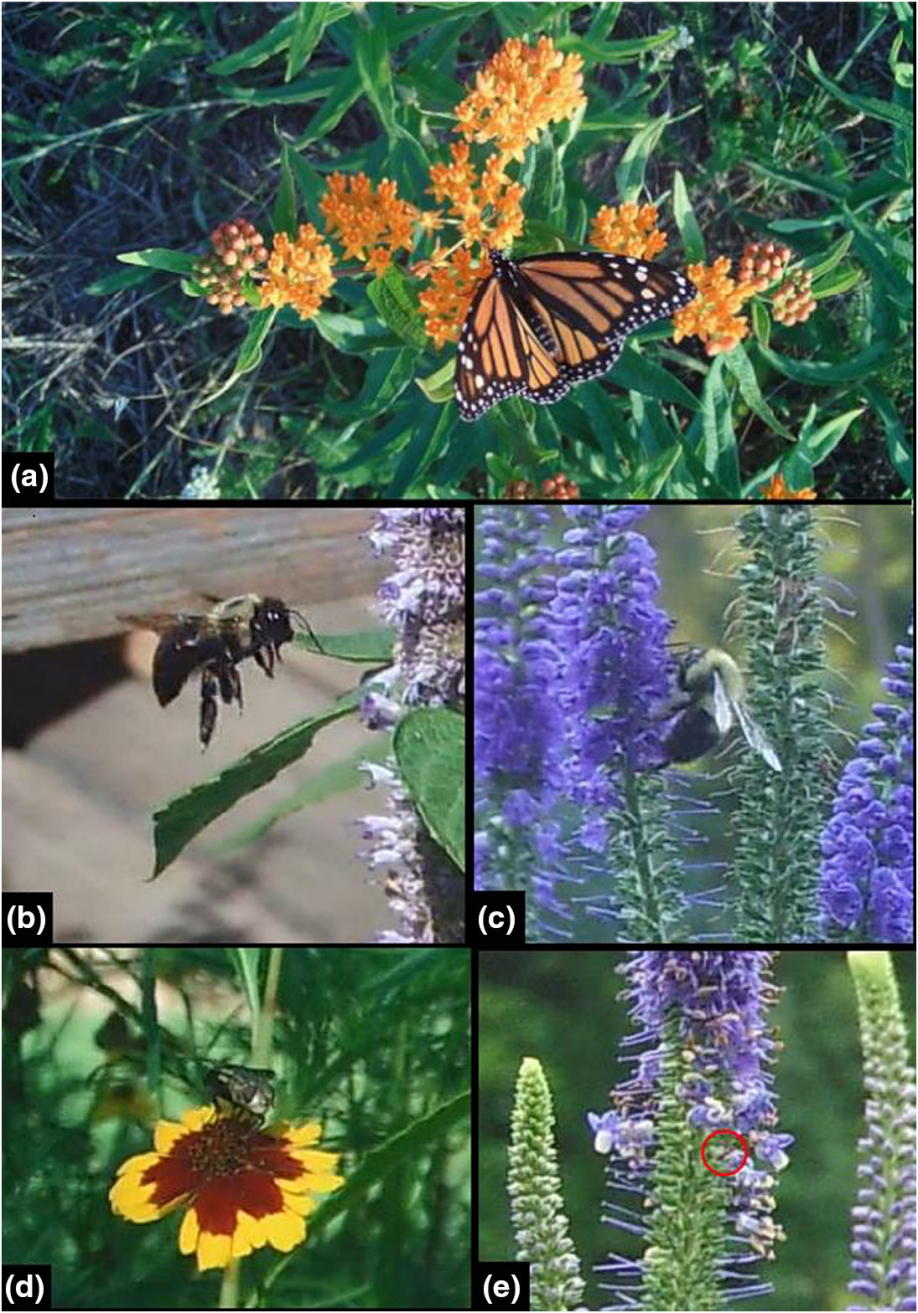
Screenshots from our game cameras displaying insects from multiple orders on or near a variety of focal flowers. Photos (b–e) are cut‐outs of larger images. (a) Monarch Butterfly (*Danaus plexippus*; order Lepidoptera) on butterfly milkweed (*Asclepias tuberosa*); (b) Carpenter Bee (*Xylocopa virginica*, order Hymenoptera) on hyssop (*Hyssopus officinalis*); (c) Bumblebee (Genus *Bombus*, order Hymenoptera) on spiked speedwell (*Veronica spicata*); (d) Fly (order Diptera) on tickseed (*Coreopsis* spp.); (e) Ant (Family Formicidae, order Hymenoptera, circled in red) on spiked speedwell (*Veronica spicata*)

We predicted that motion‐activated cameras would capture more large insects because they would be more likely to activate the motion‐sensor feature; however, scheduled cameras still outperformed motion‐activated cameras, capturing more insects of all size or age classes of Hymenoptera and Lepidoptera. A marked difference between the scheduled and motion‐activated cameras is the motion‐activated cameras’ 200 ms response time to motion triggers, which may have been too slow for the rapid movement of some flying insects and began to record when the insect was no longer in frame. This may explain in part the disparity between detection rates of motion‐activated and scheduled cameras. Due to worldwide concern over pollinator population collapse, the three‐fold increase in Hymenoptera detection by scheduled cameras compared to humans may be of interest to researchers who want to survey bee/wasp presence, richness, and inter‐ and intra‐species interactions. It should be noted, however, that identification to genus or species for many Hymenoptera is most likely not possible with game camera images (Figure 4).

Our results suggest that detection rates by the different camera settings and compared to human observations varied by insect behavior. In particular, human observers were more likely to detect insects that were exhibiting flower probing or landing on flowers whereas cameras, particularly those with motion‐activated settings, were more likely to detect insects engaged in flying or hovering behaviors. This makes intuitive sense as behaviors that result in more time at the flower (e.g., probing/landing) are more likely to be detected by the human eye whereas high motion behaviors (e.g., flying) may be more likely to be detected by motion‐activated cameras. This result would suggest that researchers should consider the type of data of interest when considering the use of cameras for monitoring plant–insect interactions.

Previous studies on insect behavior have used a variety of camera systems with adaptations or alterations that allow them to function similarly to camera traps (Droissart et al., 2021; Lortie et al., 2012; Micheneau et al., 2008; Pegoraro et al., 2020; Steen, 2017). Although effective, these systems often require some level of expertise to assemble and use. The game camera model we used required minimal setup and functioned as desired virtually “out‐of‐the‐box” with no additional alterations. Commercially available game cameras are user‐friendly and do not require technical expertise or experience to operate effectively. Additionally, the use of widely available game cameras may help improve the consistency and replicability of data.

Although game cameras present a promising tool for monitoring plant–animal interactions and behaviors, potential shortcomings should be considered when adopting this technology, with advantages and disadvantages ultimately based on specific study objectives and logistics. For example, game cameras can produce many terabytes of data that require storage and management. Following data collection, annotation of the videos and identification of insects can be time‐consuming and require taxonomic expertise. Similar to human observations on plants, there will likely be periods where there are no interactions and those “false trigger” videos still require annotation time. However, computer vision methods of automated detection and classification have the potential to alleviate the extra burden associated with processing large quantities of video data (Ratnayake et al., 2021; Weinstein, 2018). Further, game cameras do not collect video data continuously but, rather, collect video clips at set intervals or through motion‐activation. This type of data collection may prohibit certain analyses, such as the duration of foraging at a flower (e.g., Sivakoff & Gardiner, 2017). In all cases, study objectives must be carefully considered before adopting any monitoring technology.

Future studies can build upon our results by replicating our methods with different plant and insect communities and evaluating different recording schedules to optimize the quantity and quality of data collected in relation to deployment length (i.e., battery life) and study‐specific goals. For example, recording shorter videos (e.g., 10 s) may reduce the likelihood of multiple interactions being recorded within a single video clip (Steen, 2017). Plant observations are also often conducted during daylight hours (when pollinators are more visible) for set periods of time, thereby potentially missing interactions that occur at different times of day or nocturnally (Johnson et al., 2020). The night‐vision capabilities of camera traps can allow researchers to observe nocturnal insect visitation and interaction. For example, in a recent study by Sakagami et al. (2021), the nocturnal impact of ambush predators such as praying mantises on flower‐visiting moths and its plant reproductive consequences could not have been captured without cameras. The use of camera traps is key to capturing unique insect–plant interactions that are difficult to observe, and critically, to provide verifiable proof of these observations in the form of pictures or videos which can be further studied long after the interaction was captured.

Researchers and land managers often require baseline data on wildlife behavior, distribution, and abundances to make proactive land management decisions. Traditional monitoring can be time‐intensive and costly, leading to the use of technologies, such as game cameras, to augment monitoring efforts. Although game cameras have been extensively used to monitor larger species (Rovero & Zimmermann, 2016), our study demonstrates that these technologies (specifically commercially available, unaltered cameras) can also effectively be used to monitor plant‐insect interactions. Our study was designed to demonstrate the utility of this technology in a general context, but our results highlight how cameras with close‐focus lenses can facilitate data collection to address a wide number of research questions.

## AUTHOR CONTRIBUTION

**Qaim Naqvi:** Data curation (equal); Formal analysis (equal); Investigation (equal); Methodology (equal); Software (equal); Supervision (lead); Writing – original draft (lead); Writing – review & editing (lead). **Patrick Wolff:** Conceptualization (equal); Formal analysis (lead); Investigation (equal); Methodology (equal); Software (equal); Writing – original draft (equal); Writing – review & editing (equal). **Jinelle H Sperry:** Conceptualization (lead); Funding acquisition (lead); Resources (equal); Visualization (equal); Writing – review & editing (equal). **Brenda Molano‐Flores:** Formal analysis (supporting); Validation (supporting); Writing – original draft (supporting); Writing – review & editing (supporting).

## CONFLICT OF INTEREST

All authors declare that they have no conflicts of interest.

## Supporting information

Model Summaries

Video Examples

## Data Availability

Data, including video examples, model summaries, and Timelapse2 template samples available at DRYAD (https://doi.org/10.5061/dryad.q573n5tkm).
