# Supplementary material for: Camera traps are an effective tool for monitoring insect–plant interactions
Source: Ecol Evol. 2022 Jun 2;12(6):e8962. doi: 10.1002/ece3.8962 (PMC9163375; doi:10.1002/ece3.8962)
Supplement: Supplementary file 1 — Model Summaries [file ECE3-12-e8962-s002.docx]

**Supporting Information**

Generalized linear mixed models were built using the functions “glmer.nb” (for negative binomial distribution) and “glmer” (for Poisson distribution) in the package “lme4” (Bates et al., 2015).

Overdispersion was tested using the following function from Bolker et al. (2021), available at: <https://bbolker.github.io/mixedmodels-misc/glmmFAQ.html#overdispersion>

overdisp_fun <- **function**(model) {

rdf <- df.residual(model)

rp <- residuals(model,type="pearson")

Pearson.chisq <- sum(rp^2)

prat <- Pearson.chisq/rdf

pval <- pchisq(Pearson.chisq, df=rdf, lower.tail=FALSE)

c(chisq=Pearson.chisq,ratio=prat,rdf=rdf,p=pval)

}

Likelihood ratio tests were performed using the function “lrtest” in the package “lmtest” (Zeileis and Hothorn, 2002).

The following are presented for each taxonomic group: 1) variance-to-mean ratios, 2) model summary (R output) for the model using Poisson distribution, 3) dispersion test for the Poisson model, 4) zero-inflation test for the Poisson model, 5) model summary (R output) for the model using negative binomial distribution, and 6) likelihood ratio test comparing fit of the Poisson model to the negative binomial model. Overdispersion was not tested for negative binomial models, as overdispersion is irrelevant for models that estimate a scale parameter, such as the negative binomial (Bolker et al. 2021).

**References**

Bates, D., Mächler, M., Bolker, B. and Walker, S. (2015). Fitting Linear Mixed-Effects Models Usinglme4. *Journal of Statistical Software*, 67(1).

Bolker et al. (2021). GLMM FAQ. Last updated 04 Oct 2021. https://bbolker.github.io/mixedmodels-misc/glmmFAQ.html#references

Zeileis, A. and Hothorn, T. (2002). Diagnostic Checking in Regression Relationships. *R News* 2(3), 7-10. URL https://CRAN.R-project.org/doc/Rnews/

**Coleoptera**

*1)*

| Coleoptera | Mean | Variance | Ratio (Variance/Mean) |
| --- | --- | --- | --- |
| HUM | 0.4375 | 1.062500 | 2.428571 |
| MOT | 0.1875 | 0.562500 | 3.000000 |
| SCH | 0.8750 | 5.183333 | 5.923810 |

*2) Poisson model*

Generalized linear mixed model fit by maximum likelihood (Laplace Approximation) ['glmerMod']

Family: poisson ( log )

Formula: Col ~ Type + (1 | Trial_new)

Data: my_data_tax

AIC BIC logLik deviance df.resid

70.0 77.5 -31.0 62.0 44

Scaled residuals:

Min 1Q Median 3Q Max

-1.02376 -0.19238 -0.13603 -0.08905 1.71496

Random effects:

Groups Name Variance Std.Dev.

Trial_new (Intercept) 7.195 2.682

Number of obs: 48, groups: Trial_new, 16

Fixed effects:

Estimate Std. Error z value Pr(>|z|)

(Intercept) -3.5332 1.5475 -2.283 0.0224 *

TypeMOT -0.8473 0.6901 -1.228 0.2195

TypeSCH 0.6931 0.4629 1.497 0.1343

---

Signif. codes: 0 ‘***’ 0.001 ‘**’ 0.01 ‘*’ 0.05 ‘.’ 0.1 ‘ ’ 1

Correlation of Fixed Effects:

(Intr) TypMOT

TypeMOT -0.134

TypeSCH -0.199 0.447

*3) Dispersion test (p < 0.05 indicates overdispersion)*

chisq ratio rdf **p**

14.8805831 0.3381951 44.0000000 **0.9999885**

*4) Check for zero-inflation using “check_zeroinflation” function of “performance” package*

Observed zeros: 40

Predicted zeros: 40

Ratio: 1.00

Model seems ok, ratio of observed and predicted zeros is within the tolerance range.

*5) Negative binomial model*

Generalized linear mixed model fit by maximum likelihood (Laplace Approximation) ['glmerMod']

Family: Negative Binomial(78.6684) ( log )

Formula: Col ~ Type + (1 | Trial_new)

Data: my_data_tax

AIC BIC logLik deviance df.resid

72.0 81.3 -31.0 62.0 43

Scaled residuals:

Min 1Q Median 3Q Max

-1.00719 -0.19248 -0.13637 -0.08895 1.70859

Random effects:

Groups Name Variance Std.Dev.

Trial_new (Intercept) 7.181 2.68

Number of obs: 48, groups: Trial_new, 16

Fixed effects:

Estimate Std. Error z value Pr(>|z|)

(Intercept) -3.5280 1.5482 -2.279 0.0227 *

TypeMOT -0.8548 0.6964 -1.227 0.2197

TypeSCH 0.6895 0.4724 1.460 0.1444

---

Signif. codes: 0 ‘***’ 0.001 ‘**’ 0.01 ‘*’ 0.05 ‘.’ 0.1 ‘ ’ 1

Correlation of Fixed Effects:

(Intr) TypMOT

TypeMOT -0.134

TypeSCH -0.204 0.448

*6) Likelihood ratio test comparing Poisson model to negative binomial model (p < 0.05 indicates negative binomial is better fit)*

Model 1: Col ~ Type + (1 | Trial_new) negative binomial

Model 2: Col ~ Type + (1 | Trial_new) Poisson

#Df LogLik Df Chisq Pr(>Chisq)

1 5 -30.983

2 4 -31.023 -1 0.0795 **0.778**

**Diptera**

*1)*

| Diptera | Mean | Variance | Ratio (Variance/Mean) |
| --- | --- | --- | --- |
| HUM | 14.8750 | 254.3833 | 17.10140 |
| MOT | 5.9375 | 113.9292 | 19.18807 |
| SCH | 20.5625 | 648.1292 | 31.51996 |

*2) Poisson model*

Generalized linear mixed model fit by maximum likelihood (Laplace Approximation) ['glmerMod']

Family: poisson ( log )

Formula: Dip ~ Type + (1 | Trial_new)

Data: my_data_tax

AIC BIC logLik deviance df.resid

534.6 542.0 -263.3 526.6 44

Scaled residuals:

Min 1Q Median 3Q Max

-4.1865 -1.6723 -0.5917 0.8315 6.3698

Random effects:

Groups Name Variance Std.Dev.

Trial_new (Intercept) 1.782 1.335

Number of obs: 48, groups: Trial_new, 16

Fixed effects:

Estimate Std. Error z value Pr(>|z|)

(Intercept) 2.05932 0.34779 5.921 3.20e-09 ***

TypeMOT -0.91839 0.12070 -7.609 2.76e-14 ***

TypeSCH 0.32379 0.08463 3.826 0.00013 ***

---

Signif. codes: 0 ‘***’ 0.001 ‘**’ 0.01 ‘*’ 0.05 ‘.’ 0.1 ‘ ’ 1

Correlation of Fixed Effects:

(Intr) TypMOT

TypeMOT -0.099

TypeSCH -0.141 0.407

*3) Dispersion test (p < 0.05 indicates overdispersion)*

chisq ratio rdf **p**

2.768228e+02 6.291427e+00 4.400000e+01 **1.643519e-35**

*4) Check for zero-inflation using “check_zeroinflation” function of “performance” package*

Observed zeros: 10

Predicted zeros: 5

Ratio: 0.50

Model is underfitting zeros (probable zero-inflation).

*5) Negative binomial model*

Generalized linear mixed model fit by maximum likelihood (Laplace Approximation) ['glmerMod']

Family: Negative Binomial(1.053) ( log )

Formula: Dip ~ Type + (1 | Trial_new)

Data: my_data_tax

AIC BIC logLik deviance df.resid

331.9 341.3 -161.0 321.9 43

Scaled residuals:

Min 1Q Median 3Q Max

-0.9909 -0.6359 -0.2398 0.3538 1.9788

Random effects:

Groups Name Variance Std.Dev.

Trial_new (Intercept) 1.447 1.203

Number of obs: 48, groups: Trial_new, 16

Fixed effects:

Estimate Std. Error z value Pr(>|z|)

(Intercept) 2.2018 0.4196 5.248 1.54e-07 ***

TypeMOT -1.3029 0.4298 -3.031 0.00244 **

TypeSCH 0.2240 0.4163 0.538 0.59059

---

Signif. codes: 0 ‘***’ 0.001 ‘**’ 0.01 ‘*’ 0.05 ‘.’ 0.1 ‘ ’ 1

Correlation of Fixed Effects:

(Intr) TypMOT

TypeMOT -0.451

TypeSCH -0.504 0.520

*6) Likelihood ratio test comparing Poisson model to negative binomial model (p < 0.05 indicates negative binomial is better fit)*

Model 1: Dip ~ Type + (1 | Trial_new) negative binomial

Model 2: Dip ~ Type + (1 | Trial_new) Poisson

#Df LogLik Df Chisq Pr(>Chisq)

1 5 -160.95

2 4 -263.28 -1 204.66 **< 2.2e-16**

**Formicidae**

*1)*

| Formicidae | Mean | Variance | Ratio (Variance/Mean) |
| --- | --- | --- | --- |
| HUM | 1.0625 | 5.662500 | 5.329412 |
| MOT | 0.5625 | 3.195833 | 5.681481 |
| SCH | 8.1250 | 273.583333 | 33.671795 |

*2) Poisson model*

Generalized linear mixed model fit by maximum likelihood (Laplace Approximation) ['glmerMod']

Family: poisson ( log )

Formula: For ~ Type + (1 | Trial_new)

Data: my_data_tax

AIC BIC logLik deviance df.resid

132.0 139.5 -62.0 124.0 44

Scaled residuals:

Min 1Q Median 3Q Max

-1.17364 -0.36154 -0.13074 -0.09513 2.83491

Random effects:

Groups Name Variance Std.Dev.

Trial_new (Intercept) 8.49 2.914

Number of obs: 48, groups: Trial_new, 16

Fixed effects:

Estimate Std. Error z value Pr(>|z|)

(Intercept) -2.7374 1.0754 -2.545 0.0109 *

TypeMOT -0.6360 0.4122 -1.543 0.1228

TypeSCH 2.0343 0.2579 7.889 3.05e-15 ***

---

Signif. codes: 0 ‘***’ 0.001 ‘**’ 0.01 ‘*’ 0.05 ‘.’ 0.1 ‘ ’ 1

Correlation of Fixed Effects:

(Intr) TypMOT

TypeMOT -0.133

TypeSCH -0.212 0.553

*3) Dispersion test (p < 0.05 indicates overdispersion)*

chisq ratio rdf **p**

22.8649467 0.5196579 44.0000000 **0.9964738**

*4) Check for zero-inflation using “check_zeroinflation” function of “performance” package*

Observed zeros: 34

Predicted zeros: 32

Ratio: 0.94

Model is underfitting zeros (probable zero-inflation).

*5) Negative binomial model*

Generalized linear mixed model fit by maximum likelihood (Laplace Approximation) ['glmerMod']

Family: Negative Binomial(231.299) ( log )

Formula: For ~ Type + (1 | Trial_new)

Data: my_data_tax

AIC BIC logLik deviance df.resid

133.8 143.2 -61.9 123.8 43

Scaled residuals:

Min 1Q Median 3Q Max

-1.16461 -0.36053 -0.13027 -0.09478 2.76602

Random effects:

Groups Name Variance Std.Dev.

Trial_new (Intercept) 8.526 2.92

Number of obs: 48, groups: Trial_new, 16

Fixed effects:

Estimate Std. Error z value Pr(>|z|)

(Intercept) -2.7468 1.0991 -2.499 0.0125 *

TypeMOT -0.6361 0.4153 -1.531 0.1256

TypeSCH 2.0364 0.2628 7.748 9.3e-15 ***

---

Signif. codes: 0 ‘***’ 0.001 ‘**’ 0.01 ‘*’ 0.05 ‘.’ 0.1 ‘ ’ 1

Correlation of Fixed Effects:

(Intr) TypMOT

TypeMOT -0.132

TypeSCH -0.212 0.551

*6) Likelihood ratio test comparing Poisson model to negative binomial model (p < 0.05 indicates negative binomial is better fit)*

Model 1: For ~ Type + (1 | Trial_new) negative binomial

Model 2: For ~ Type + (1 | Trial_new) Poisson

#Df LogLik Df Chisq Pr(>Chisq)

1 5 -61.901

2 4 -62.010 -1 0.2182 **0.6404**

**Hemiptera**

*1)*

| Hemiptera | Mean | Variance | Ratio (Variance/Mean) |
| --- | --- | --- | --- |
| HUM | 0.2500 | 0.600000 | 2.400000 |
| MOT | 0.3125 | 1.029167 | 3.293333 |
| SCH | 15.5000 | 3649.733333 | 235.466667 |

*2) Poisson model*

Generalized linear mixed model fit by maximum likelihood (Laplace Approximation) ['glmerMod']

Family: poisson ( log )

Formula: Hem ~ Type + (1 | Trial_new)

Data: my_data_tax

AIC BIC logLik deviance df.resid

86.6 94.1 -39.3 78.6 44

Scaled residuals:

Min 1Q Median 3Q Max

-1.4495 -0.0509 -0.0072 -0.0065 8.2433

Random effects:

Groups Name Variance Std.Dev.

Trial_new (Intercept) 46.8 6.841

Number of obs: 48, groups: Trial_new, 16

Fixed effects:

Estimate Std. Error z value Pr(>|z|)

(Intercept) -9.9579 2.4927 -3.995 6.47e-05 ***

TypeMOT 0.2231 0.6708 0.333 0.739

TypeSCH 4.1271 0.5040 8.189 2.64e-16 ***

---

Signif. codes: 0 ‘***’ 0.001 ‘**’ 0.01 ‘*’ 0.05 ‘.’ 0.1 ‘ ’ 1

Correlation of Fixed Effects:

(Intr) TypMOT

TypeMOT -0.150

TypeSCH -0.199 0.739

*3) Dispersion test (p < 0.05 indicates overdispersion)*

chisq ratio rdf **p**

1.287171e+02 2.925389e+00 4.400000e+01 **3.081933e-10**

*4) Check for zero-inflation using “check_zeroinflation” function of “performance” package*

Observed zeros: 41

Predicted zeros: 43

Ratio: 1.05

Model seems ok, ratio of observed and predicted zeros is within the tolerance range.

*5) Negative binomial model*

Generalized linear mixed model fit by maximum likelihood (Laplace Approximation) ['glmerMod']

Family: Negative Binomial(25.4374) ( log )

Formula: Hem ~ Type + (1 | Trial_new)

Data: my_data_tax

AIC BIC logLik deviance df.resid

87.7 97.1 -38.9 77.7 43

Scaled residuals:

Min 1Q Median 3Q Max

-1.4416 -0.0513 -0.0078 -0.0073 6.9402

Random effects:

Groups Name Variance Std.Dev.

Trial_new (Intercept) 46.24 6.8

Number of obs: 48, groups: Trial_new, 16

Fixed effects:

Estimate Std. Error z value Pr(>|z|)

(Intercept) -9.7220 2.5162 -3.864 0.000112 ***

TypeMOT 0.1484 0.7247 0.205 0.837781

TypeSCH 3.9078 0.5683 6.876 6.14e-12 ***

---

Signif. codes: 0 ‘***’ 0.001 ‘**’ 0.01 ‘*’ 0.05 ‘.’ 0.1 ‘ ’ 1

Correlation of Fixed Effects:

(Intr) TypMOT

TypeMOT -0.158

TypeSCH -0.229 0.701

*6) Likelihood ratio test comparing Poisson model to negative binomial model (p < 0.05 indicates negative binomial is better fit)*

Model 1: Hem ~ Type + (1 | Trial_new) negative binomial

Model 2: Hem ~ Type + (1 | Trial_new) Poisson

#Df LogLik Df Chisq Pr(>Chisq)

1 5 -38.866

2 4 -39.323 -1 0.9139 **0.3391**

**Hymenoptera**

*1)*

| Hymenoptera | Mean | Variance | Ratio (Variance/Mean) |
| --- | --- | --- | --- |
| HUM | 48.250 | 6248.20 | 129.4964 |
| MOT | 112.125 | 43764.92 | 390.3226 |
| SCH | 207.625 | 90700.25 | 436.8465 |

*2) Poisson model*

m01.Hym.poisson <- glmer(Hym ~ Type + (1|Trial_new), data=my_data_tax, family = "poisson")

Generalized linear mixed model fit by maximum likelihood (Laplace Approximation) ['glmerMod']

Family: poisson ( log )

Formula: Hym ~ Type + (1 | Trial_new)

Data: my_data_tax

AIC BIC logLik deviance df.resid

1814.8 1822.3 -903.4 1806.8 44

Scaled residuals:

Min 1Q Median 3Q Max

-8.9120 -2.9232 -0.8844 4.1343 17.6745

Random effects:

Groups Name Variance Std.Dev.

Trial_new (Intercept) 2.054 1.433

Number of obs: 48, groups: Trial_new, 16

Fixed effects:

Estimate Std. Error z value Pr(>|z|)

(Intercept) 2.86163 0.36118 7.923 2.32e-15 ***

TypeMOT 0.84322 0.04301 19.603 < 2e-16 ***

TypeSCH 1.45934 0.03993 36.549 < 2e-16 ***

---

Signif. codes: 0 ‘***’ 0.001 ‘**’ 0.01 ‘*’ 0.05 ‘.’ 0.1 ‘ ’ 1

Correlation of Fixed Effects:

(Intr) TypMOT

TypeMOT -0.083

TypeSCH -0.090 0.753

*3) Dispersion test (p < 0.05 indicates overdispersion)*

chisq ratio rdf **p**

1.388998e+03 3.156814e+01 4.400000e+01 **2.306759e-262**

*4) Check for zero-inflation using “check_zeroinflation” function of “performance” package*

Observed zeros: 4

Predicted zeros: 0

Ratio: 0.00

Model is underfitting zeros (probable zero-inflation).

*5) Negative binomial model*

Generalized linear mixed model fit by maximum likelihood (Laplace Approximation) ['glmerMod']

Family: Negative Binomial(0.8311) ( log )

Formula: Hym ~ Type + (1 | Trial_new)

Data: my_data_tax

AIC BIC logLik deviance df.resid

502.0 511.4 -246.0 492.0 43

Scaled residuals:

Min 1Q Median 3Q Max

-0.9009 -0.5536 -0.3047 0.4715 1.9584

Random effects:

Groups Name Variance Std.Dev.

Trial_new (Intercept) 1.7 1.304

Number of obs: 48, groups: Trial_new, 16

Fixed effects:

Estimate Std. Error z value Pr(>|z|)

(Intercept) 3.29589 0.44130 7.469 8.1e-14 ***

TypeMOT -0.06386 0.47151 -0.135 0.89227

TypeSCH 1.20897 0.42847 2.822 0.00478 **

---

Signif. codes: 0 ‘***’ 0.001 ‘**’ 0.01 ‘*’ 0.05 ‘.’ 0.1 ‘ ’ 1

Correlation of Fixed Effects:

(Intr) TypMOT

TypeMOT -0.469

TypeSCH -0.486 0.480

*6) Likelihood ratio test comparing Poisson model to negative binomial model (p < 0.05 indicates negative binomial is better fit)*

Model 1: Hym ~ Type + (1 | Trial_new) negative binomial

Model 2: Hym ~ Type + (1 | Trial_new) Poisson

#Df LogLik Df Chisq Pr(>Chisq)

1 5 -246.01

2 4 -903.38 -1 1314.7 **< 2.2e-16 *****

**Lepidoptera**

*1)*

| Lepidoptera | Mean | Variance | Ratio (Variance/Mean) |
| --- | --- | --- | --- |
| HUM | 1.4375 | 7.195833 | 5.005797 |
| MOT | 0.3750 | 1.183333 | 3.155556 |
| SCH | 7.7500 | 487.933333 | 62.959140 |

*2) Poisson model*

Generalized linear mixed model fit by maximum likelihood (Laplace Approximation) ['glmerMod']

Family: poisson ( log )

Formula: Lep ~ Type + (1 | Trial_new)

Data: my_data_tax

AIC BIC logLik deviance df.resid

159.4 166.9 -75.7 151.4 44

Scaled residuals:

Min 1Q Median 3Q Max

-2.2837 -0.3870 -0.1667 -0.0851 7.1260

Random effects:

Groups Name Variance Std.Dev.

Trial_new (Intercept) 7.218 2.687

Number of obs: 48, groups: Trial_new, 16

Fixed effects:

Estimate Std. Error z value Pr(>|z|)

(Intercept) -2.2499 0.9809 -2.294 0.02180 *

TypeMOT -1.3437 0.4584 -2.932 0.00337 **

TypeSCH 1.6848 0.2270 7.422 1.16e-13 ***

---

Signif. codes: 0 ‘***’ 0.001 ‘**’ 0.01 ‘*’ 0.05 ‘.’ 0.1 ‘ ’ 1

Correlation of Fixed Effects:

(Intr) TypMOT

TypeMOT -0.097

TypeSCH -0.195 0.418

*3) Dispersion test (p < 0.05 indicates overdispersion)*

chisq ratio rdf **p**

1.032010e+02 2.345477e+00 4.400000e+01 **1.163451e-06**

*4) Check for zero-inflation using “check_zeroinflation” function of “performance” package*

Observed zeros: 34

Predicted zeros: 32

Ratio: 0.94

Model is underfitting zeros (probable zero-inflation).

*5) Negative binomial model*

Generalized linear mixed model fit by maximum likelihood (Laplace Approximation) ['glmerMod']

Family: Negative Binomial(0.822) ( log )

Formula: Lep ~ Type + (1 | Trial_new)

Data: my_data_tax

AIC BIC logLik deviance df.resid

141.2 150.6 -65.6 131.2 43

Scaled residuals:

Min 1Q Median 3Q Max

-0.8089 -0.3850 -0.2326 -0.0885 3.3252

Random effects:

Groups Name Variance Std.Dev.

Trial_new (Intercept) 5.765 2.401

Number of obs: 48, groups: Trial_new, 16

Fixed effects:

Estimate Std. Error z value Pr(>|z|)

(Intercept) -1.6109 1.0187 -1.581 0.1138

TypeMOT -1.5224 0.8416 -1.809 0.0705 .

TypeSCH 1.1392 0.6171 1.846 0.0649 .

---

Signif. codes: 0 ‘***’ 0.001 ‘**’ 0.01 ‘*’ 0.05 ‘.’ 0.1 ‘ ’ 1

Correlation of Fixed Effects:

(Intr) TypMOT

TypeMOT -0.230

TypeSCH -0.417 0.450

*6) Likelihood ratio test comparing Poisson model to negative binomial model (p < 0.05 indicates negative binomial is better fit)*

Model 1: Lep ~ Type + (1 | Trial_new)

Model 2: Lep ~ Type + (1 | Trial_new)

#Df LogLik Df Chisq Pr(>Chisq)

1 5 -65.615

2 4 -75.717 -1 20.202 **6.967e-06**
